# Reconstructive Approaches in Surgical Management of Congenital Pseudarthrosis of the Tibia

**DOI:** 10.3390/jcm9124132

**Published:** 2020-12-21

**Authors:** Andrea Laufer, Adrien Frommer, Georg Gosheger, Robert Roedl, Frank Schiedel, Jan Niklas Broeking, Gregor Toporowski, Anna Rachbauer, Carina Antfang, Bjoern Vogt

**Affiliations:** 1 Pediatric Orthopedics, Deformity Reconstruction and Foot Surgery, University Hospital Muenster, 48149 Muenster, Germany; andrea.laufer@ukmuenster.de (A.L.); adrien.frommer@ukmuenster.de (A.F.); robert.roedl@ukmuenster.de (R.R.); janniklas.broeking@ukmuenster.de (J.N.B.); gregor.toporowski@ukmuenster.de (G.T.); anna.rachbauer@ukmuenster.de (A.R.); carina.antfang@ukmuenster.de (C.A.); 2 General Orthopedics and Tumor Orthopedics, University Hospital Muenster, 48149 Muenster, Germany; georg.gosheger@ukmuenster.de; 3 Pediatric Orthopedics and Deformity Reconstruction, Clemens Hospital Muenster, 48153 Muenster, Germany; f.schiedel@alexianer.de

**Keywords:** congenital pseudarthrosis of the tibia, bone transport, distraction osteogenesis, external fixator, intramedullary nail

## Abstract

Treatment of congenital pseudarthrosis of the tibia remains a major challenge in pediatric orthopedics. Ideal timing and preference of surgical procedures are discussed controversially. A variety of reconstructive treatment strategies have been described in literature, but so far none has proven its superiority. The aim of treatment is to obtain long-term bone union, to prevent refracture, and to correct angular deformities and leg length discrepancies. This study retrospectively evaluates the outcome of different reconstructive strategies. Sixty-nine patients were identified who presented to our outpatient department between 1997 and 2019. Twenty-six of these patients underwent reconstructive surgical treatment and were included in this study. The study cohort was divided into three groups. Excision of the pseudarthrosis was performed in all patients in Group A and B, and in two patients of Group C. Group A (six/26 patients) received subsequent bone transport through external fixation maintaining original length. In Group B (15/26 patients), patients underwent either previous, simultaneous, or subsequent extrafocal lengthening through external fixation to reconstitute length. In Group C (five/26 patients), internal fixation with intramedullary nails was applied. Radiological and clinical evaluation was performed to assess bone union and complication rates. Results varied considerably between the study groups. Overall, the primary bone fusion rate was 69.2%. There were four refractures, all occurring in Group B. The long-term bone union rate without refracture was 53.8%. The overall complication rate was 53.8% and 23.1% showed persistent pseudarthrosis. Two secondary amputations were performed due to failed bone fusion. In conclusion, excision of the pseudarthrosis and extrafocal lengthening achieves a satisfying bone union rate and limb reconstruction, while bone transport does not offer significant advantages but shows higher complication rates. Intramedullary stabilization should be applied to maintain bone union, but shows lower bone union rates when used as a stand-alone treatment regimen. Regardless of the primary bone fusion rates, the probability of long-term bone union remains unpredictable.

## 1. Introduction

**Figure 1:**
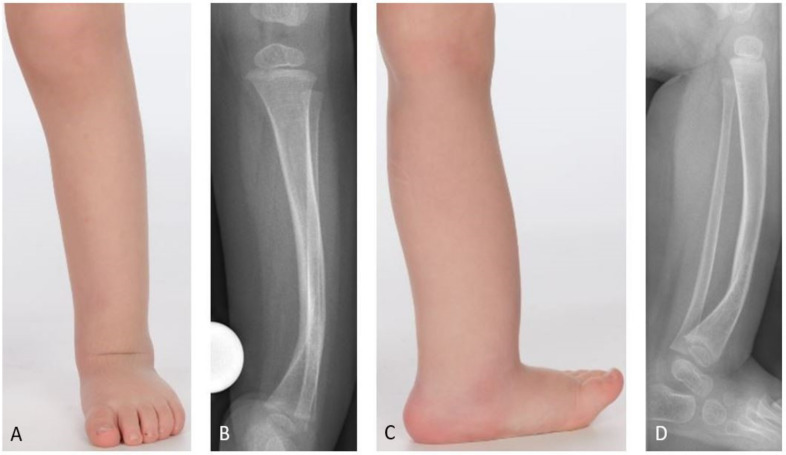
Clinical and radiological presentation of anterolateral bowing in congenital pseudarthrosis of the tibia (CPT); (**A**,**B**) anterior-posterior (a.p.) view; (**C**,**D**) lateral view.

Congenital pseudarthrosis of the tibia (CPT) is a rare condition of unclear etiology [1]. The estimated prevalence of association with neurofibromatosis (NF) type 1 is 55%, even though van Royen et al. recently indicated that the true prevalence might be significantly higher (up to 84%) [2,3,4,5,6]. In most cases the pseudarthrosis is not yet present at birth, but the tibia shows a characteristic anterolateral bowing which deteriorates during growth (Figure 1). A fracture frequently develops spontaneously or after a minor trauma. This leads to a pseudarthrosis with atrophy of the bone ends and a surrounding soft tissue hamartoma impeding physiological healing [7,8,9]. The loss of remodeling potential and the growth inhibition of the tibia lead to further deterioration of the deformity and to leg length discrepancy (LLD) [7].

It is assumed that for compensation of LLD, the ipsilateral femur reacts with overgrowth, which can lead to coxa valga [1,8,10]. The shortening of the affected lower leg frequently causes insufficiency of the gastrocnemius and soleus muscles, resulting in pes calcaneus [8,11]. The fibula is additionally affected in about 50% of the cases [11]. Pseudarthrosis of the fibula often causes proximal migration of the fibula, subsequently producing progressive ankle valgus.

**Figure 2:**
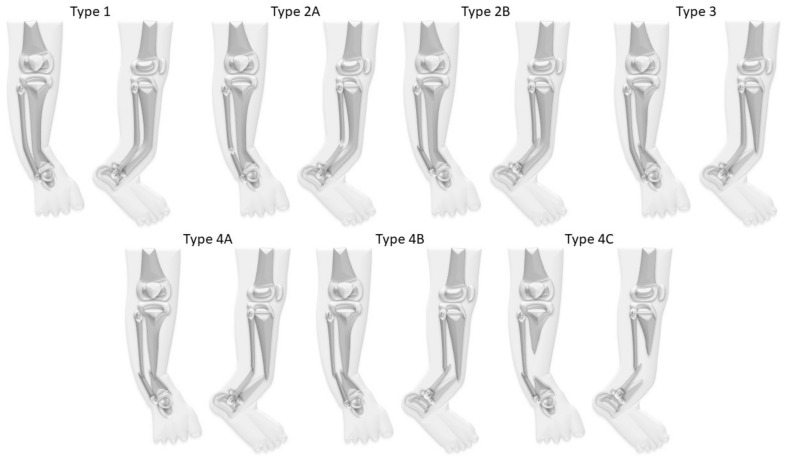
Paley classification for CPT. Type 1: No fractures. Type 2A: No fracture of the tibia; fractured fibula, the fibula is not dislocated. Type 2B: No fracture of the tibia; fractured and proximally migrated fibula. Type 3: Fractured tibia, no fracture of the fibula. Type 4A: Tibia and fibula both fractured. Type 4B: Tibia and fibula both fractured, the fibula is migrated proximally. Type 4C: Bone defect of the tibia, the fibula is migrated proximally [7].

While the Andersen, Boyd, and Crawford classifications are of a rather descriptive nature, the El-Rosasy-Paley classification, which was introduced in 2007, also considers previous unsuccessful surgery [4,9,12,13]. Paley introduced another classification in 2019 which emphasizes the importance of the fibula for treatment and outcome (Figure 2) [8].

### Treatment Approaches

Even though there is no general consensus on the ideal timing for surgery, most authors restrain from surgical interventions in children under the age of three years, but recommend conservative treatment instead [7,8]. Orthotic bracing for external stabilization of the leg should be fitted when the child starts weight-bearing [7]. The aim is to prevent further progression of the deformity; ideally, the occurrence of a fracture can be avoided [8,14]. Surgery is commonly recommended in case of a fracture, as spontaneous healing is unlikely [7,8]. Surgical treatment aims to gain and maintain bone union and a satisfactory functional outcome [8,11]. Nevertheless, even after successful primary bone union secondary complications—in particular, refracture of the pseudarthrosis—are common and often require further surgeries [1].

Excision of the fibrous hamartoma and pathological periosteum is supposed to be the key surgical procedure to achieve primary bone union [2,15]. To constitute bone fusion, multiple reconstructive treatment strategies based on different fixation techniques have been described [7,8]. Vascularized fibula transfer is a reconstructive approach that has shown satisfying bone union rates particularly in large tibial bone defects, but has the disadvantage of donor site morbidity [16,17,18]. The cross-union concept—e.g., through ‘4-in-1 osteosynthesis’—has recently been established as a stand-alone treatment regimen, aiming to create a cross-fusion between tibia and fibula in order to achieve stable bone union and prevent refracture [8,19]. Bone union rates seem to be very promising, but results are still preliminary.

With the introduction of the Ilizarov technique, external fixation has become increasingly popular, allowing simultaneous gradual correction of LLD and limb malalignment [7,20]. The Ilizarov technique has shown acceptable rates of primary bone union in the past and additionally bears the advantage of repetitive applications, unlike, e.g., vascularized fibular bone grafting [7,21,22]. Depending on the Ilizarov technique, acute compression and extrafocal tibial lengthening as well as bone transport have proven to be successful reconstructive treatment approaches to achieve bone fusion [1,7,23,24]. When bone union is obtained, it is commonly recommended to further maintain external stabilization through orthotic fitting to prevent refracture [7]. Additionally, some authors propose internal support of the bone, e.g., through intramedullary rodding [7,25].

Amputation should be reserved for cases of failed reconstruction; the decision for amputation should preferably be made by a center providing sufficient expertise in treatment of this rare disease [8].

So far, none of the prevalent reconstructive methods has proven its superiority. This study evaluates the outcome of three different reconstructive strategies for treatment of CPT focusing on bone fusion rates, secondary complications, and outcome.

## 2. Patients and Methods

**Figure 3:**
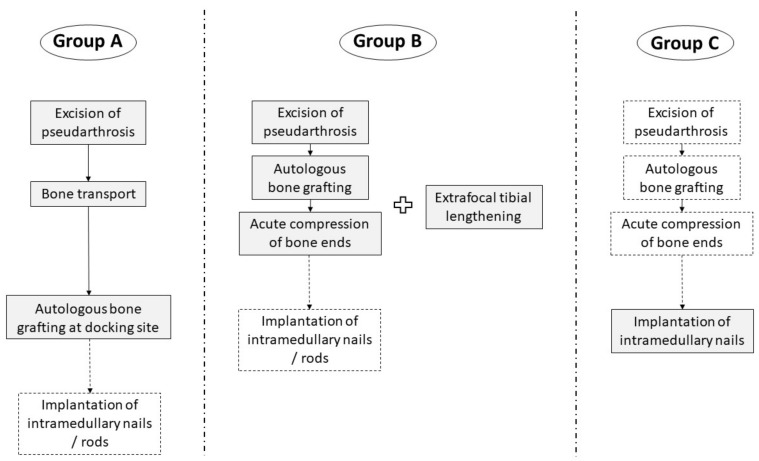
Surgical procedures in the different study groups. Boxes with dashed lines: procedures were not performed in all patients.

A single-center cohort of 69 patients (33 (48%) females, 36 (52%) males) with CPT treated between 1997 and 2019 was investigated retrospectively. Twelve patients (17%) did not receive further treatment after initial consultation and were lost to follow-up. Two patients (3%) underwent primary amputation on their own request due to previous failed surgery. Fifteen (22%) were treated conservatively, and 40 (58%) received surgical treatment. Fourteen patients underwent minor surgical interventions, mostly for growth modulation. Twenty-six patients received reconstructive surgery in regard of treatment of the pseudarthrosis, and were included in the study. The study cohort was divided into three subgroups based on the reconstructive procedure which had been performed (Figure 3). Types of CPT were classified according to Paley (Figure 1, Table 1, Table 2 and Table 3).

In Group A (*n* = 6, 23%), surgical treatment consisted of excision of the pseudarthrosis and fibrous hamartoma and extrafocal corticotomy at the proximal tibia, subsequently followed by bone transport with diaphyseal transfer through external fixation. Autologous bone grafting was performed secondary at the docking site. Thus, the original length of the lower leg was maintained. For bone transport, an Ilizarov frame was used in two patients, and a Taylor Spatial Frame^TM^ (TSF^TM^, Smith+Nephew, Watford, UK) in four patients. Intramedullary stabilization through K-wires was applied in two patients.

In Group B (*n* = 15, 58%), autologous bone grafting was performed after excision of the pseudarthrosis, before inducing acute compression of the bone ends, resulting in shortening of the lower leg. Extrafocal proximal tibial lengthening by distraction osteogenesis using external fixation was either done previously (*n* = 3) or simultaneously (*n* = 12) to reconstitute length. For distraction osteogenesis, a circular external fixator (TSF^TM^) was used in 14 cases, and a unilateral rail system (Limb Reconstruction System^TM^ (LRS^TM^), Orthofix Medical Inc., Lewisville, TX, USA) in one case. After removal of the external fixator, nine patients (60%) received intramedullary stabilization to prevent refracture, either through implantation of intramedullary rods (*n* = 7; MK medical, Emmingen-Liptingen, Germany) or a Fassier-Duval telescoping nail (*n* = 2; Pega Medical Inc., Laval, QC, Canada).

In Group C (*n* = 5, 19%), previous excision of the pseudarthrosis and acute compression of the bone ends was performed in two patients; additional bone grafting at the resection site was implemented in one of them. A Fassier-Duval telescopic intramedullary nail was implanted in skeletally immature patients, and a TRIGEN^TM^ intramedullary trauma nail (Smith+Nephew, Watford, UK) in patients who had already attained skeletal maturity. Loss of leg length in case of pseudarthrosis resection was compensated through orthotic braces.

For autologous bone grafting, corticospongious bone was harvested either from the ipsi- or contralateral iliac crest. Adjuncts to surgical intervention, such as topical application of bone morphogenetic protein (BMP) or bisphosphonates (BP) prior to surgery, were not applied.

After removal of the external fixator, long-leg plaster cast was applied for another six weeks, followed by orthotic bracing (custom-made ankle-foot orthoses (AFO)), which was kept until skeletal maturity was attained.

In Groups A and B, the following parameters of distraction osteogenesis and bone transport were evaluated:

- Distraction distance in mm,
- Time with external fixation in days,
- Distraction index: Time with external fixation divided through total distraction distance (days/cm) [21],
- Consolidation time in months,
- Consolidation index: Time from end of distraction to bony union in months, divided by the amount of lengthening or transport in centimeters (months/cm) [25].

Complications occurring during distraction and consolidation period were classified into minor (no requirement for or only minor additional surgery) and major complications (either requiring corrective surgery or negatively affecting the surgical outcome) [25].

A modified version of the evaluation criteria introduced by Johnston in 2002 was applied to classify radiological and clinical results [26]:

- Grade 1:
  ◦ Radiologically: Full bone union (compulsory);
  ◦ Clinically: Pain-free full weight-bearing (with or without orthotic bracing), physiological alignment primarily maintained or restored; no requirement for further surgery.
- Grade 2:
  ◦ Radiologically: Inadequate bone union (residual transverse or longitudinal cortical defect; bone atrophy; insufficient bone remodeling/circumferential cortical thickening);
    and/or
  ◦ Clinically: Persistent or recurrent limb malalignment (severe valgus/sagittal bowing) requiring further surgery.
- Grade 3:
  ◦ Radiologically: Persistent non-union or recurrent fracture (compulsory);
  ◦ Clinically: Painful mobilization, instable lower leg.

This is a retrospective cohort study with no inferential statistics. Descriptive statistics were employed to summarize the study outcome.

The study was approved by the independent ethics committee of the University of Muenster on 1 July 2019 (registration number: 2019-368-f-S) and conducted according to the principles of the World Medical Association Declaration of Helsinki.

## 3. Results

**Figure 4:**
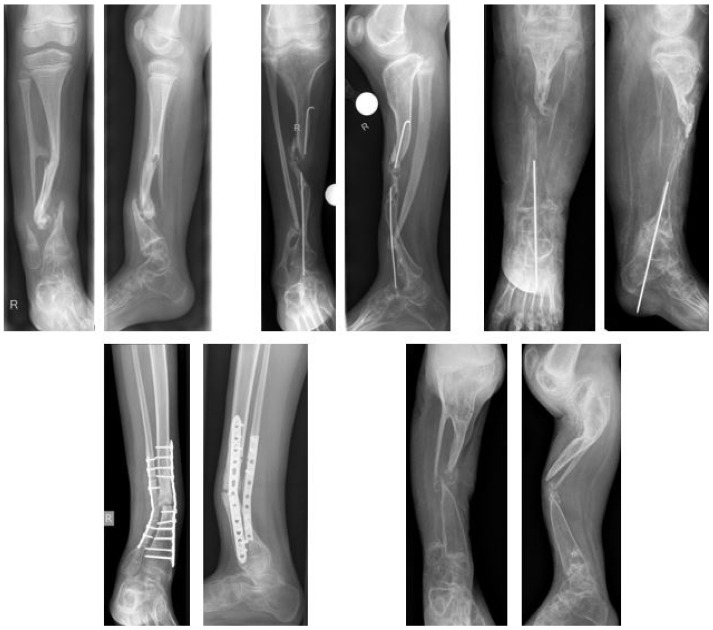
Examples of previous failed surgeries (a.p. and lateral views).

In 29 of 69 patients with CPT (42%) an association with NF type 1 was observed. In the study cohort, NF type I was present in 12 of 26 patients (46%). Even though, the true prevalence might have been higher, as a molecular genetic analysis was not initiated in all patients. There were 16 females (62%) and ten males (39%). More than two thirds of patients included (69%) had undergone previous surgeries with a mean of 2.6 (1–7) interventions, mostly performed in external institutions (Figure 4). The mean age at first surgical intervention at the study center was 8.7 (2.8–14.9) years; only one child included in the study was younger than three years. Eight patients (31%) had not received previous surgical treatment; these patients had a mean age of 6.5 (2.8–11.0) years at the time of the index procedure.

Mean follow-up was 7.4 (1.0–20.0) years; a minimum follow-up of two years was not achieved in two patients. Thirteen patients (50%) had attained skeletal maturity at the time of last follow-up.

In the study cohort CPT was classified according to Paley as follows: Paley type 1 in three patients (12%), type 2A in one patient (4%), type 3 in four patients (15%), type 4A in 16 patients (62%), type 4B in one patient (4%), and type 4C in one patient (4%).

**Figure 5:**
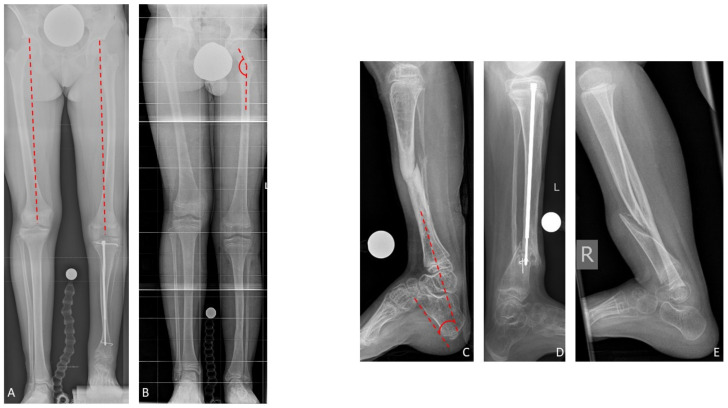
Associated deformities: Femoral overgrowth (**A**), coxa valga with increased neck-shaft angle (**B**), and pes calcaneus with decreased tibiocalcaneal angle (**C**–**E**).

Nine of 19 patients (47%) of whom radiographs of both femora were available presented femoral overgrowth with a femoral segment which was ≥10.0 mm longer than the contralateral femur. All of them additionally showed coxa valga. In total, 17 of 21 patients (81%) with radiographs of the ipsilateral femur presented coxa valga. The mean neck-shaft angle in the study cohort was 148.1 (130–164) degrees on the leg affected by CPT, and 132.9 (119–149) on the contralateral side. Pes calcaneus was present in 16 of 24 patients (67%) in whom the tibiocalcaneal angle could be evaluated. Pes calcaneus was defined by a tibiocalcaneal angle below 60 degrees. Overall, the mean tibiocalcaneal angle measured 55.8 (23–78) degrees on the leg affected by CPT (Figure 5). The contralateral tibiocalcaneal angle could not be evaluated as lateral radiographs of the contralateral leg were not available.

### 3.1. Radiological and Functional Outcome

#### 3.1.1. Bone Union Rates

Primary bone union was achieved in 18 of 26 patients (69%), although this rate varied considerably between the three different groups. Bone fusion was observed in average 6.9 (1–20) months after termination of reconstructive treatment (after removal of the external fixator or after implantation of the intramedullary nail). In Groups A and B, mean distraction distance measured 58.6 (30–137) mm; the mean distraction index was 42.1 (26.1–57.8) days/cm and mean consolidation index was 1.0 (0.1–3.7) months/cm. Overall, there were four refractures (22% refracture rate). Though all refractures occurred in Group B, in average 4.8 (1–8) years after primary bone union had been achieved. Long-term bone union without refracture was achieved in a total of 14 (54%) patients.

Overall, according to the modified Johnston criteria there were 11 results grade 1 (42%), three results grade 2 (12%), and twelve results grade 3 (46%).

In 12 patients presenting NF type I, primary bone fusion was achieved in nine patients (75%), while this was the case in nine of 14 patients (64%) without NF type I. In patients with previous surgeries, primary bone union rate was 78%, and long-term bone union rate 67%. In those patients for whom the investigated treatment was the index procedure, primary bone union could be obtained in four (50%), and long-term bone union in two patients (25%). Three patients were younger than six years at the time of index procedure, of whom long-term bone union could be achieved in one. Overall, six patients (23%) were younger than six years old at the time of reconstructive surgery, of whom long-term bone union could be attained in three (50%). Of 20 patients (77%) who were older than six years, long-term bone union could be achieved in 11 (55%).

There were eight cases (31%) of failed primary bone union, of which three (38%) achieved secondary bone union after further reconstructive surgery. In six of 26 patients (23%), permanent bone fusion was not achieved (persistent pseudarthrosis). Two secondary amputations were performed due to failed bone union, one in Group A and one in Group B.

Twenty-two patients of the study cohort (85%) received a mean of 2.4 (1–5) subsequent surgeries after initial reconstruction, mostly for lengthening by distraction osteogenesis.

#### 3.1.2. Functional Outcome

Seventeen of 21 patients (81%) in whom evaluation of leg length was available showed residual LLD of in average 4.3 (0.9–11.0) cm at the time of last follow-up. Regarding adjacent joint deformities, nine patients (35%) presented ankle fusion, and five patients (19%) presented severe ankle valgus. Five patients (29%) showed no limitations concerning movement of the ankle joint after termination of treatment. Distal tibiofibular cross-union was observed in five cases, of whom two had received tibiofibular transfixation through fixation screws.

At the time of last follow-up, all patients were ambulating and able to bear full weight without pain, but 14 patients (54%) required orthotic bracing (AFO): All patients with persistent or recurrent pseudarthrosis, and eight patients in whom long-term bone union had been achieved, but who showed adjacent deformities such as severe ankle valgus requiring external stabilization. Patients presenting bone union who had achieved skeletal maturity did not receive further orthotic support. The two patients who had been amputated were able to ambulate in exoprosthesis.

### 3.2. Results Group A: Excision of the Pseudarthrosis and Bone Transport

The mean age at first surgery was 8.6 (6.4–12.3) years. The initial LLD prior to reconstruction was in average 8.0 (2–12) cm. The average tibial resection length was 8.5 (5.5–13) cm. Mean period of external fixation was 306.2 (244–434) days. Mean time from application of the fixator to bone docking was 177.6 (105–341) days. Mean distraction distance measured 67.8 (50–103) mm. Mean distraction index was 48.8 (37.9–57.8) days/cm and mean consolidation index was 1.1 (1.0–1.1) months/cm. After removal of the external fixator, intramedullary rods for internal bone stabilization were implemented in two patients (33%).

Primary bone union was achieved in three patients (50% primary bone union rate). There were no refractures (0% refracture rate), thus the long-term bone union rate was also 50%. All but one patient received further surgeries, which ultimately led to secondary bone union in one of three patients with failed primary bone union (33% secondary bone union rate). Secondary amputation due to failed bone union was performed in one patient, and one patient showed persistent pseudarthrosis at the time of last follow-up.

**Figure 6:**
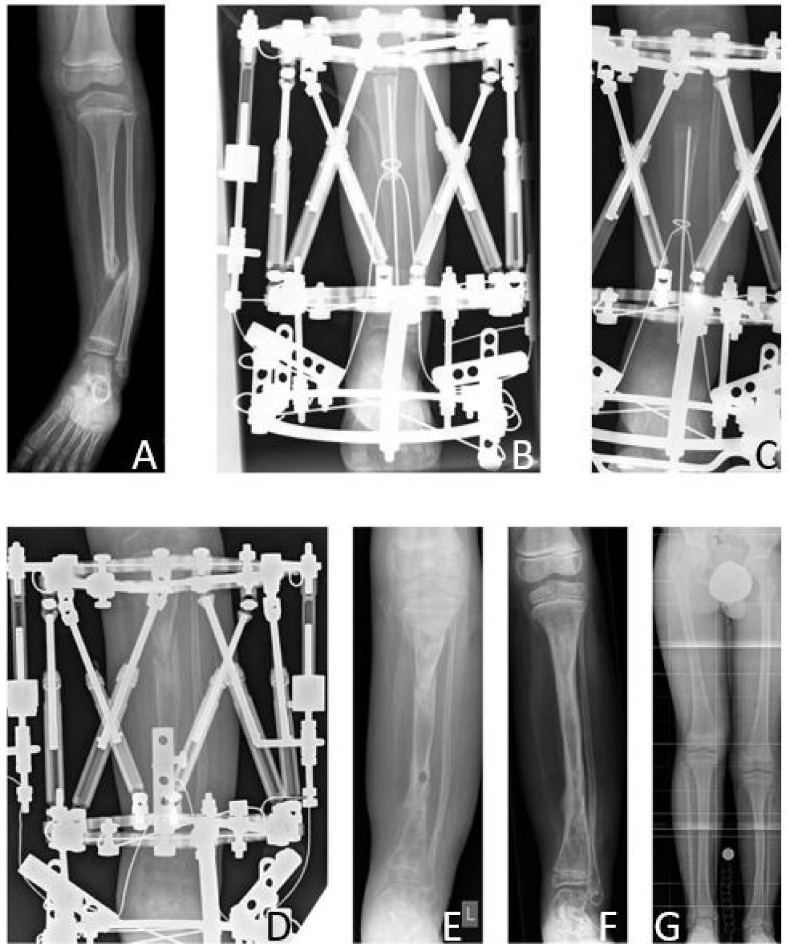
Results Group A. Seven-year-old male patient presenting CPT Paley type 3 (**A**). Bone transport was performed over a total distance of 50 mm (**B**,**C**). After bone docking (**D**) the external fixator was applied for another three months. After removal of the fixator (**E**), full bone consolidation was observed after 10 months (**F**). Five years after reconstruction, at the age of 13 years, the patient showed a remaining LLD of 2.8 cm (**G**).

According to the modified Johnston criteria, there was one result grade 1, two grade 2, and three grade 3 (Figure 6).

### 3.3. Results Group B: Excision of the Pseudarthrosis, Acute Compression, and Extrafocal Lengthening

The mean age at first surgery was 9.2 (3.7–14.9) years. The initial LLD was in average 4.4 (0–11.5) cm. Mean tibial resection length measured 4.3 (3–6) cm. Mean period of external fixation was 219.1 (151–441) days. Mean distraction distance was 54.8 (30–137) mm. Mean distraction index was 39.9 (26.1–57.7) days/cm, and mean consolidation index was 1.0 (0.1–3.7) months/cm.

Intramedullary rods were implanted in nine patients (60%) after termination of reconstructive treatment.

Primary bone union was achieved in 12 patients (80% primary bone union rate). Four refractures were observed in the former pseudarthrosis site (33% refracture rate). Two of four refractures occurred despite intramedullary stabilization (2.5 mm intramedullary rod (MK medical, Emmingen-Liptingen, Germany) and 4.8 mm Fassier-Duval telescoping nail, respectively). Of seven patients who had either not achieved primary bone union or suffered a refracture, secondary bone fusion was achieved in two (29% secondary bone union rate). Eight patients achieved long-term bone union without refracture (53% long-term bone union rate). Secondary amputation due to failed bone fusion was performed in one patient.

**Figure 7:**
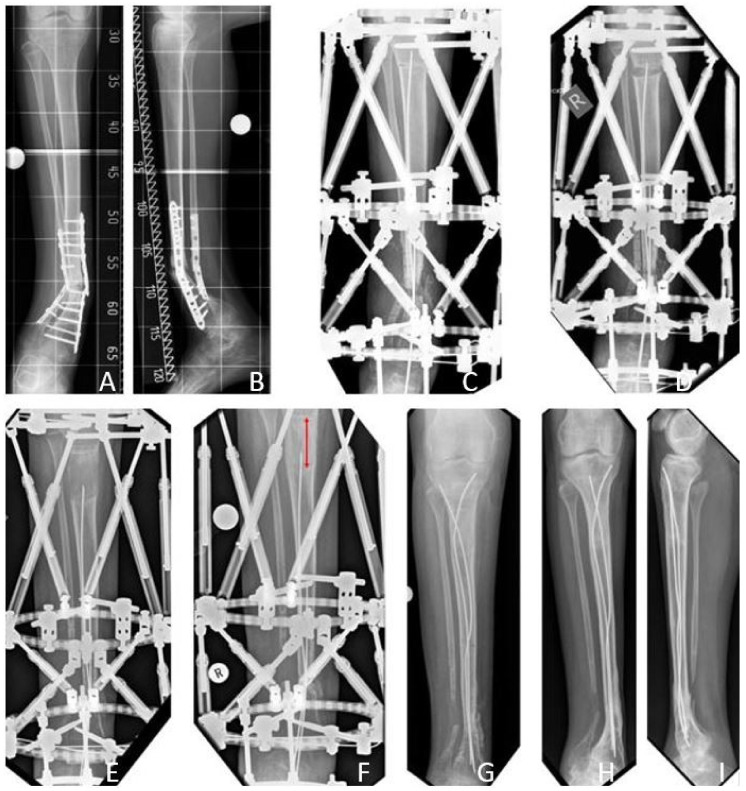
Results Group B. Fifteen-year-old male patient presenting CPT Paley type 4A with failed previous surgeries (**A**: a.p. view; **B**: lateral view). After excision of 5 cm and acute compression (**C**), distraction osteogenesis of 57 mm was performed (**D**,**E**). The bone regenerate showed satisfying consolidation after six months (**F**: red arrow: bone regenerate). Intramedullary rods were implanted when the TSF^TM^ was removed (**G**). At the time of last follow-up five years after reconstruction there was no sign of refracture, but progressive ankle valgus (**H**: a.p. view; **I**: lateral view).

Regarding the modified Johnston criteria, seven results were grade 1, one grade 2, and seven grade 3 (Figure 7).

### 3.4. Results Group C: Intramedullary Nailing

The mean age at first surgery was 7.1 (2.8–14.2) years. Excision of the pseudarthrosis and acute compression before intramedullary stabilization was performed in two cases, while three patients solely received intramedullary nailing for stabilization of the pseudarthrosis site. Four patients (80%) were treated with a Fassier-Duval telescoping nail, and one patient (20%) with a TRIGEN^TM^ intramedullary nail. Postoperatively a cast was applied for six weeks. Primary bone union was achieved in three patients (60% primary bone union rate). Excision of the pseudarthrosis had been performed in one of the two cases of failed primary bone union. There were no refractures (0% refracture rate), thus the long-term bone union rate was also 60%. Both patients who failed to achieve primary bone union neither achieved secondary bone union, and showed persistent pseudarthrosis at the time of last follow-up.

**Figure 8:**
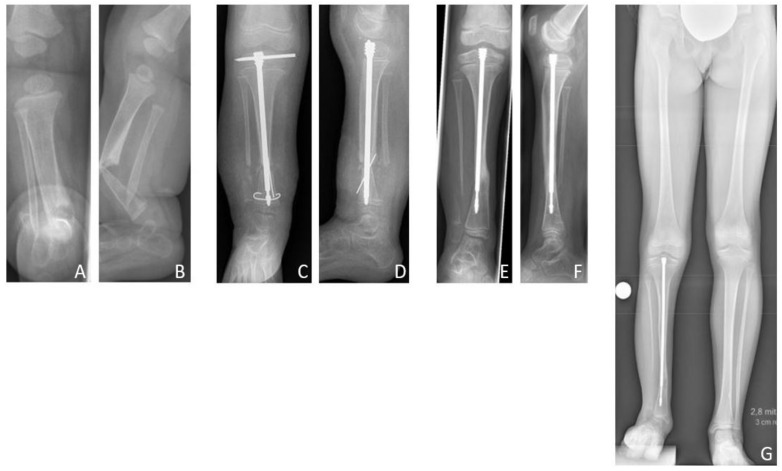
Results Group C. Six-month-old female patient with CPT, presenting fracture and pseudarthrosis of the right tibia (**A**,**B**). After conservative treatment with orthotic braces, first surgery with resection of the pseudarthrosis and implantation of a Fassier-Duval telescoping nail was performed at the age of four years (**C**,**D**). Radiologically, consolidation was achieved 14 months postoperatively (**E**,**F**). The patient received subsequent distraction osteogenesis for lengthening of 5 cm when six years old. At the time of last follow-up at the age of nine years she presented a residual LLD of 3.4 cm and ankle valgus (**G**). (a.p. and lateral views.).

According to the modified Johnston criteria, three results were grade 1 and two grade 3 (Figure 8).

### 3.5. Complications

Overall, 14 complications occurred during or after reconstructive treatment (54% complication rate). There were seven minor and seven major complications.

In Group A, there were three major and one minor complications (67%): One distal femoral fracture which occurred during bone transport, two cases of premature bony consolidation and malfunction of the bone transport mechanism requiring surgical intervention; and one case of dislocation of fixation screws.

In Group B, complication rate was 47% with two major and five minor complications. There were two distal femoral fractures occurring during distraction, requiring adaption of the external fixation to stabilize the fracture. Regarding minor complications, there were four cases of pin site infections, and one dislocation of a fixation screw.

In Group C, there were two major and one minor complications (60%): One case of infection of the Fassier-Duval nail two years after implantation and one dislocation of the female part of the Fassier-Duval nail, both requiring surgical intervention; and one case of dislocation of the K-wire locking the Fassier-Duval nail distally. Bone union had not yet been achieved when the Fassier-Duval nail had to be removed; [14] at the time of last follow-up, the patient showed persistent pseudarthrosis but was able to walk in orthotic braces.

## 4. Discussion

Treatment of CPT remains challenging, and to date there is no universal consensus on the ideal timing and procedure to gain bone union [7,8,11,14]. In surgical treatment, excision of the pseudarthrosis is generally recommended, but multiple fixation techniques aiming to achieve and maintain bone union exist [8,11,14]. Vascularized fibular bone grafting, intramedullary nailing with bone grafting, and the Ilizarov technique are supposed to be the primary surgical approaches that provide the best results. Vascularized fibula transfer has shown varying primary bone union rates of 38% to 93% but has been reported as a successful technique in treatment of large tibial defects, thus presenting an alternative to bone transport [18,27]. However, it bears the disadvantage of donor site morbidity—in particular, development of ankle valgus—and is not applicable a second time in case of recurrent pseudarthrosis. Treatment approaches such as the ‘4-in-1 osteosynthesis’ described by Choi et al. and the cross-union method introduced by Paley aim to achieve a partial fusion of tibia and fibula, thus creating stable bone union and preventing refracture [8,19]. Both techniques have shown very promising preliminary results, but long-term bone union rates and eventual functional outcome will have to be further investigated as the concept itself is relatively new and patient numbers are small.

The mean primary bone union rate in the present study was approximately 70%. Nevertheless, the extrafocal lengthening group, which included a considerably larger number of patients and therefore should be evaluated separately, showed a primary bone union rate of 80%. But even if primarily union of the pseudarthrosis is achieved, the rate of secondary complications remains high, in particular the occurrence of refracture of the pseudarthrosis site [8]. In his review of the literature, Paley stated in 2019 that, regardless of the chosen treatment, overall a union of the pseudarthrosis without refracture can be obtained in only about 50% of the cases [8]. This study supports this assumption, as long-term bone union without refracture was achieved in only 53%. Most patients included had undergone previous surgical interventions. Even though, in general, previous failed surgical interventions are supposed to negatively affect treatment outcome, this observation was not made in the present study [8,11,28]. Patients with prior surgeries showed considerably higher bone union rates than patients for whom the reconstructive procedure investigated was the index procedure, both in regard of primary as well as long-term bone union. Association with NF type 1 is also described as a negative prognostic factor for outcome of surgical treatment of CPT by some authors [11]. However, this supposition could neither be confirmed by the results of this study, since patients with NF type 1 seemed to show better bone union rates in comparison to patients without NF type 1.

The overall refracture rate in the present study was 22%, though all refractures occurred in Group B (33%). Review of the literature shows extremely variable refracture rates. In studies with patient numbers of more than 20, refracture rates of 0–40% are reported for the Ilizarov technique in combination with intramedullary rodding, and 15–61% for the Ilizarov technique without rodding [15,21,29,30,31,32]. Thus, according to the literature review, the probability for the occurrence of refracture after reconstructive treatment with the Ilizarov technique seems to be lower if additionally internal stabilization by intramedullary rodding is applied [23]. Residual or recurrent malalignment (in particular, ankle valgus) with consecutive stress risers, young patient age at index procedure, and non-compliance with orthotic bracing are also supposed to be important factors increasing the risk of refracture [22,25,33]. In the present study the numbers of refractures in patients with and without intramedullary stabilization—either Fassier-Duval telescoping nail or intramedullary rods—were equal, but the total number of only four refractures does not allow a valid statistical analysis of the accompanying factors.

Regarding the best timing to perform reconstructive treatment, some authors suggest operating before the age of three years to maintain maximal residual growth of the tibia [34,35]. Most authors, however, recommend postponing the first surgical intervention to an older age [2,7,21,24,28,33,36]. In the present study, there seemed to be no strong correlation between age of the patient at the time of reconstructive treatment and eventual outcome. However, it is assumed that the longer the follow-up period after reconstruction until skeletal maturity, the more likely the occurrence of secondary complications and refracture [8,33]. Therefore, the authors generally refrain from performing surgery in patients younger than three years; instead, conservative treatment is resumed as long as possible.

Agashe et al. in 2012 recommended to perform bone transport in case of LLD of more than 2.5 cm, and acute compression if LLD was less than 2.5 cm [25]. In the present study, subject to the different group sizes, extrafocal lengthening seemed to provide better results regarding union of the pseudarthrosis site and equalization of leg length at a lower complication rate. Nevertheless, the initial LLD was considerably higher in the bone transport group than in the extrafocal lengthening group, which supports the general assumption of Agashe et al. that bone transport should be considered in case of increased initial LLD. Reconstructive treatment with intramedullary nails as a stand-alone procedure, on the other hand, has the disadvantage of not allowing correction of LLD, which has to be performed secondary.

All patients in the study group were able to bear full weight without pain after termination of reconstructive treatment, even if long-term bone union had not been achieved; even though, orthotic braces were required by more than half of all patients. Functional outcome regarding the ankle joint remains poor. Only 30% showed free range of motion of the ankle joint; one third showed ankle fusion, and approximately 20% showed progressive ankle valgus. While patients presenting ankle fusion generally have no difficulties to ambulate if adequate orthotic braces are provided, severe ankle valgus leads to instability of the ankle joint and consecutively decreased mobility.

### Current Treatment Regimen

In consequence of their findings, the authors have now made extrafocal lengthening and subsequent intramedullary stabilization their primary treatment approach. Bone transport, however, remains an alternative choice of treatment in extensive bone defects which do not allow acute compression. In case of persistent or recurrent LLD, extrafocal lengthening can be repeated at a later point of time, if applicable even with an internal lengthening device. Internal stabilization through implantation of intramedullary rods or telescoping nails after termination of reconstruction is applied in all patients to prevent refracture. Intramedullary nailing as a stand-alone treatment regimen is considered in patients presenting only moderate bone defects, deformities, and LLD. As a fracture or pseudarthrosis of the fibula can lead to instability of the ankle joint and progressive ankle valgus, and thus consecutively increase the risk of refracture of the tibia, distal tibiofibular fixation is performed in all patients presenting fibula deficiency to achieve tibiofibular cross-union [7,8,19]. The use of adjunctive procedures such as application of BMP or BP has become increasingly popular in the past two decades, showing encouraging preliminary results with satisfying bone union rates [7,37,38,39]. Nevertheless, neither BMP nor BP is currently applied by the authors as the use in children is off-label in Germany. Apart from that, prospective multicenter trials—in particular, investigating effectiveness and potential side effects such as malignant transformation or growth abnormalities—are still due [14,31,40].

The present study has several limitations. First of all, only half of the patients included had attained skeletal maturity at the time of last follow-up. Thus, further investigations in the future might show higher rates of secondary complications such as refractures. Furthermore, due to the heterogeneity of the study groups, in particular regarding number of patients included, a statistically significant evaluation can hardly be implemented. Even though numerical tendencies indicate that specific accompanying factors such as number of previous surgeries, presence or absence of NF type I, or age at index surgery may not be as crucial as generally assumed. Nevertheless, every single case should be assessed individually, and it remains difficult to give a prediction about the eventual success of treatment.

## 5. Conclusions

In reconstructive treatment of CPT, excision of the pseudarthrosis and acute compression followed by extrafocal lengthening and subsequent intramedullary stabilization achieves satisfying results regarding bone union rates. Bone transport, on the other hand, shows a higher complication rate, but should be preserved for cases of extensive bone defects. Maintenance of long-term bone union without refracture remains difficult, but accompanying factors such as previous failed surgeries might be less crucial than generally assumed.

## Figures and Tables

**Table 1:** Results Group A: Excision of the pseudarthrosis and bone transport.

Patient No.	Sex	NF Type 1	Previous Surgeries	Paley Type	Age at First Surgery (Years)	Primary Bone Union	Refracture	Johnston Grade	Remaining LLD (cm)	Subsequent Surgeries	Follow-up (Years)	Skeletal Maturity	Outcome
1	F	No	1	1	6.4	Yes	No	2	0.0	2	17.8	Yes	Ankle valgus
2	M	No	3	4C	11.8	No	-	3	-	1	2.5	No	Amputation
3	M	Yes	1	4A	12.3	No	-	3	7.0	2	5.1	Yes	No limitations
4	M	No	0	4A	7.4	No	-	3	n/a	2	5.4	No	Persistent pseudarthrosis
5	F	Yes	2	4A	6.9	Yes	No	2	11.0	2	5.0	No	No limitations
6	M	Yes	0	3	6.9	Yes	No	1	2.8	0	7.8	No	Ankle fusion

Patient data. M = male, F = female. n/a = not available.

**Table 2:** Results Group B: Excision of the pseudarthrosis, acute compression and extrafocal lengthening.

Patient No.	Sex	NF Type 1	Previous Surgeries	Paley Type	Age at first Surgery (Years)	Primary Bone Union	Refracture	Johnston Grade	Remaining LLD (cm)	Subsequent Surgeries	Follow-up (Years)	Skeletal Maturity	Outcome
1	M	No	0	1	7.4	Yes	Yes	3	6.5	5	5.9	No	Persistent pseudarthrosis
2	M	Yes	4	4A	14.9	Yes	No	1	0.0	0	5.7	Yes	Ankle valgus
3	F	No	2	4A	13.5	Yes	No	1	3.5	4	6.9	Yes	Ankle fusion
4	F	No	2	4A	8.9	Yes	No	1	10.5	4	5.2	Yes	Ankle fusion
5	F	No	2	4A	6.3	Yes	Yes	3	0.0	4	9.2	Yes	Ankle fusion
6	F	No	1	4A	13.2	Yes	No	1	0.0	0	2.6	Yes	Ankle fusion
7	F	Yes	3	2A	7.9	Yes	No	2	0.9	2	20.0	Yes	Ankle valgus
8	F	No	0	3	3.7	No	-	3	5.1	4	6.4	No	Ankle fusion
9	F	No	1	4A	4.9	Yes	No	1	0.0	2	10.1	Yes	No limitations
10	M	Yes	7	4A	7.8	Yes	Yes	3	3.4	0	8.5	Yes	Persistent pseudarthrosis
11	M	Yes	5	4A	11.8	No	-	3	0.0	1	1.0	No	Persistent pseudarthrosis
12	F	Yes	2	4A	5.0	No	-	3	-	1	1.0	No	Amputation
13	F	Yes	2	4A	13.9	Yes	No	1	4.0	2	10.9	Yes	No limitations
14	F	Yes	0	4A	11.0	Yes	Yes	3	4.5	4	11.1	Yes	Ankle fusion
15	M	No	2	4A	7.7	Yes	No	1	1.5	2	6.0	No	Ankle valgus

Patient data. M = male, F = female. n/a = not available.

**Table 3:** Results Group C: Excision of the pseudarthrosis, acute compression, and intramedullary nailing.

Patient No.	Sex	NF Type 1	Previous Surgeries	Paley Type	Age at first Surgery (Years)	Primary Bone Union	Refracture	Johnston Grade	Remaining LLD (cm)	Subsequent Surgeries	Follow-up (Years)	Skeletal Maturity	Outcome
1	F	No	0	4B	4.4	Yes	No	1	3.5	3	3.9	No	Ankle valgus
2	F	Yes	1	3	6.0	Yes	No	1	1.0	2	4.0	No	No limitations
3	M	No	0	3	8.2	No	-	3	n/a	1	2.6	No	Persistent pseudarthrosis
4	F	Yes	4	1	14.2	Yes	No	1	1.5	1	8.3	Yes	Ankle fusion
5	F	No	0	4A	2.8	No	-	3	1.9	2	3.4	No	Persistent pseudarthrosis

Patient data. M = male, F = female. n/a = not available.
